# Data on multiple SARS-CoV-2 surface glycoprotein alignments

**DOI:** 10.1016/j.dib.2021.107414

**Published:** 2021-09-23

**Authors:** Done Stojanov

**Affiliations:** Faculty of Computer Science, Goce Delčev University of Štip, Krste Misirkov No.10-A P.O. Box 201, Štip 2000, North Macedonia

**Keywords:** SARS-CoV-2, Surface glycoprotein, Computational analysis, Variants, Mutations

## Abstract

Severe acute respiratory syndrome coronavirus 2 (SARS-CoV-2) surface glycoproteins deposited to the NCBI GenBank from Europe, by the mid of April 2021 (12.04.2021) were analysed. At least one amino acid mutation relative to YP_009724390.1 referent SARS-CoV-2 surface glycoprotein: Wuhan-Hu-1 complete genome (NCBI accession: NC_045512) was found in 788 SARS-CoV-2 surface glycoproteins. Data was computed by NCBI Cobalt multiple alignment tool [1] (one country by another) and structured by special purpose application developed in Visual Studio 2019. Advanced data structures were used to organize computed data. Linked dataset lists program output: *SARS-CoV-2 surface glycoprotein mutations per processed protein*.

## Specifications Table

**Table:** 

Subject	Computer Science, Bioinformatics, Computational Biology, Genetics: General
Specific subject area	Data on SARS-CoV-2 surface glycoprotein alignments in Europe
Type of data	Tables in Microsoft Excel Workbook (.xlsx)
How the data were acquired	NCBI virus database (https://www.ncbi.nlm.nih.gov/labs/virus/vssi/#/) was accessed to acquire accession identifiers of analysed SARS-CoV-2 surface glycoproteins. Google Chrome Browser on HP notebook with AMD Ryzen 7 3700U with Radeon Vega Mobile Gfx 2.30 GHz, 8 GB RAM, 64-bit operating system - Windows 10 Enterprise was used to access data.
Data format	Analysed
Parameters for data collection	NCBI virus database: https://www.ncbi.nlm.nih.gov/labs/virus/vssi/#/ accessed Filters applied: •Virus: Severe acute respiratory syndrome coronavirus 2 •taxid: 2697049 •Sequence Type: GenBank •Nucleotide Completeness: complete •Proteins: surface glycoprotein •Geographic Region: Europe •Collection dates: 11.04.2021 and 12.04.2021
Description of data collection	NCBI virus database was accessed to retrieve identifiers (IDs) of SARS-CoV-2 spike proteins. Partial protein annotations were not considered. Proteins with unknown amino acid due to unknown nucleotide(s) in S genes were not aligned. NCBI Cobalt multiple alignment tool was used to compute amino acid mutations. Cobalt parameters selected: •Gap penalties: -2,-1 •End-Gap penalties: -2,-1 •Use RPS BLAST: on •Blast E-value: 0.003 •Find Conserved columns and Recompute: on •Use query clusters: on •Word Size: 3 •Max cluster distance: 0.8 •Alphabet: Regular
Data source location	•Institution: The National Center for Biotechnology Information •City/Town/Region: Bethesda, Maryland •Country: The United States of America
Data accessibility	Data is included in this article
Related research article	D. Stojanov Phylogenicity of B. 1.1. 7 surface glycoprotein, novel distance function and first report of V90T missense mutation in SARS-CoV-2 surface glycoprotein, Meta Gene, 30 (2021), pp. 100967, https://doi.org/10.1016/j.mgene.2021.100967[2]

## Value of the Data

• Reliable phylogenetic networks can be constructed based on the available data. Knowing SARS-CoV-2 spike protein mutations is useful for automated prediction of the change in binding affinity towards neutralizing antibodies. Many crystallographic structures of neutralizing antibodies in complex to SARS-CoV-2 spike protein domains (such as: N-terminal domain (NTD), receptor-binding domain (RBD)) are available at Protein Data Bank (https://www.rcsb.org/) and they can serve as templates to predict updates in binding affinity upon SARS-CoV-2 spike protein mutations.
• Provided data can be exploited to analyse SARS-CoV-2 surface glycoprotein phylogenesis. Also suitable for mutagenetic studies. Changes in protein stability, protein flexibility and protein to protein interactions upon computed alterations, can be computationally inspected with available tools, such as: DynaMut (http://biosig.unimelb.edu.au/dynamut/) [3], Duet (http://biosig.unimelb.edu.au/duet/) [4], SDM (http://marid.bioc.cam.ac.uk/sdm2) [5], mCSM-PPI2 (http://biosig.unimelb.edu.au/mcsm_ppi2/) [6].
• Evolutionary properties of SARS-CoV-2 surface glycoprotein can be monitored based on provided data. Conclusions for enhanced (reduced) variants’ infectivity due to SARS-CoV-2 spike protein mutations can be suggested.

## 1 Data Description

Appendix A available in [2], lists computed SARS-CoV-2 surface glycoprotein variants. Combination of mutations on protein level specifies variant. Data on each SARS-CoV-2 spike protein variant is arranged as: {listofmutations,Hits:N}, such as: listofmutations is a text-form representation of computed amino acid mutations and N equals the number of SARS-CoV-2 spike proteins matching specific variant. XpY annotation (amino acid X was changed to amino acid Y at position p) is used to mark substitution and Xp− annotation marks deletion (amino acid X at position p was deleted ‘−‘). Given that at least one SARS-CoV-2 spike protein with unique combination of mutations is required to report a new variant, then N≥1.

Data shown in Appendix A in [2] was computed from bigger and detailed dataset, available in this article. Microsoft Excel Workbook named SARS-CoV-2SpikeProteinMutations.xlsx contains report on mutations found in each SARS-CoV-2 surface glycoprotein. The workbook contains 18 sheets, one sheet per country. Sheets contain: metadata, mutations report and statistics.

Metadata provides information on:

• **Country**: origin of SARS-CoV-2 surface glycoproteins;
• **Date of accession**: date of GenBank accession;
• **Number of SARS-CoV-2 spike proteins with unknown amino acids (at least one)**: if any, their number is reported here;
• **Number of SARS-CoV-2 spike proteins (no unknown amino acid)**: refers to SARS-CoV-2 surface glycoproteins with at least one mutation relative to YP_009724390.1 referent SARS-CoV-2 surface glycoprotein (Wuhan-Hu-1 complete genome, NCBI accession: NC_045512);
• **Date of computation**: the date when alignment was performed;
• **Sequences with unknown amino acid**: listed by accession identifiers.

Following metadata section, mutations report is provided. Mutations report lists SARS-CoV-2 spike proteins with at least one amino acid change relative to YP_009724390.1 spike reference. For each SARS-CoV-2 spike protein there is sperate entry provided. There are two attributes per entry:

• **Seq: ID:** NCBI accession identifier of SARS-CoV-2 spike protein;
• **Changes:** List of computed mutations in SARS-CoV-2 spike protein.

Annotation (X→Y)p:P marks that amino acid X was changed to amino acid Y at position P, while (X→−)p:P marks amino acid X deletion at position P.

Statistics on the type and number of mutations is also provided.

**Fig. 1:**
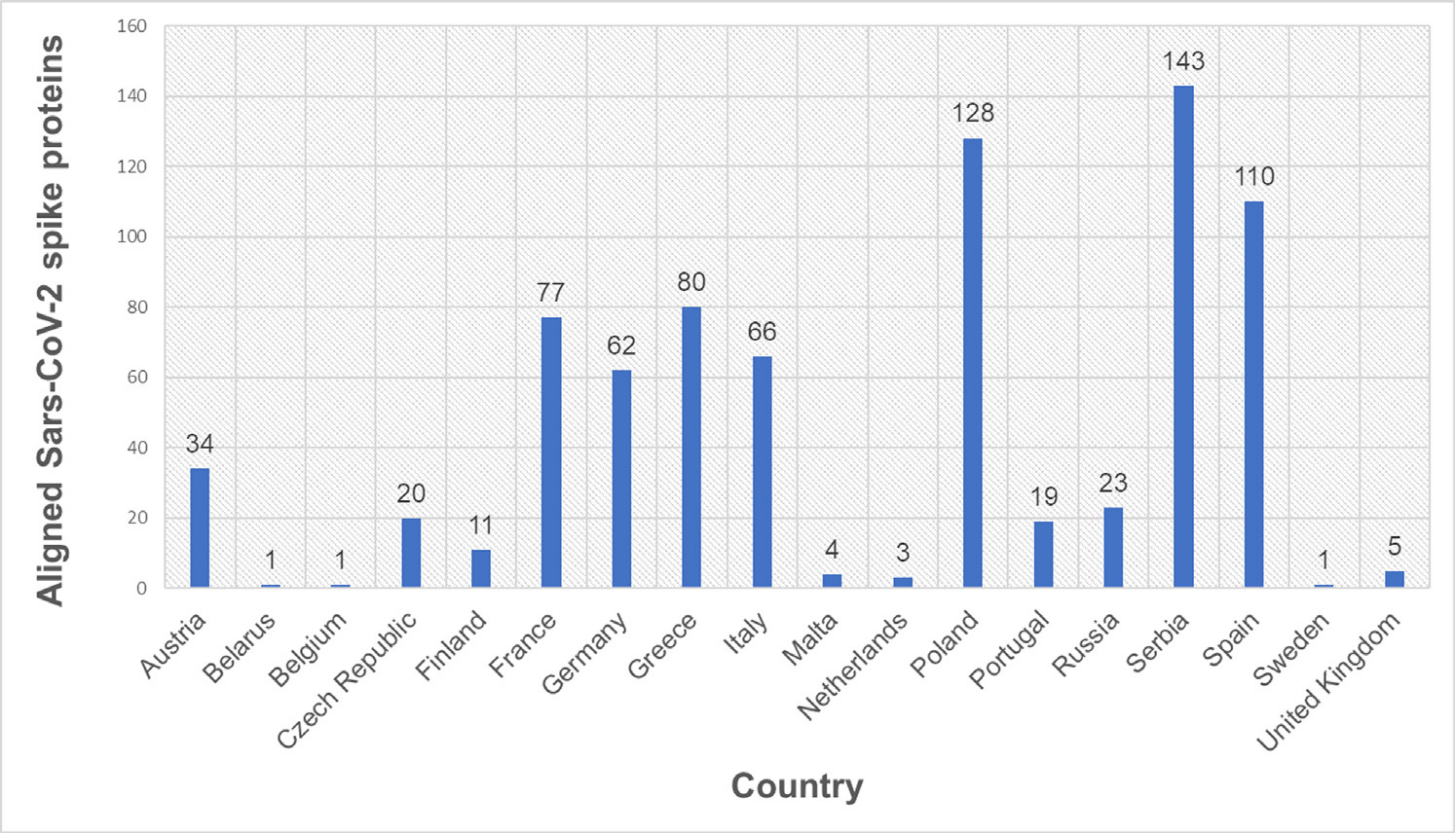
Aligned SARS-CoV-2 spike proteins per country.

Microsoft Excel Workbook SARS-CoV-2SpikeProteinMutations.xlsx, available in this article, contains data on mutations found in 788 SARS-CoV-2 spike proteins. SARS-CoV-2 spike proteins that matched exactly the referent protein (YP_009724390.1) are not reported in the dataset. None of the spike proteins that contained unknown amino acid and there were 28 such (Table 1) was aligned to the referent protein. The number of aligned SARS-CoV-2 spike proteins per country and the type/number of computed mutations, also per country, are listed in Table 1, Fig. 1 and Table 2.

**Table 1:** Number of SARS-CoV-2 spike proteins per country.

	Accession and	SARS CoV-2 spike	Aligned
	Computation	proteins with unknown	Sars-CoV-2
Country	date	amino acid	spike proteins
Austria	11.04.2021	1	34
Belarus	11.04.2021	0	1
Belgium	11.04.2021	0	1
Czech Republic	11.04.2021	3	20
Finland	11.04.2021	0	11
France	11.04.2021	0	77
Germany	11.04.2021	5	62
Greece	11.04.2021	0	80
Italy	12.04.2021	5	66
Malta	12.04.2021	0	4
Netherlands	12.04.2021	1	3
Poland	12.04.2021	5	128
Portugal	12.04.2021	3	19
Russia	12.04.2021	0	23
Serbia	12.04.2021	2	143
Spain	12.04.2021	2	110
Sweden	12.04.2021	0	1
United Kingdom	12.04.2021	1	5
**TOTAL**		**28**	**788**

**Table 2:** Type/Number of mutations per country.

	Accession and	Amino acid	Amino acid
Country	Computation date	substitutions	deletions
Austria	11.04.2021	196	72
Belarus	11.04.2021	3	0
Belgium	11.04.2021	1	14
Czech Republic	11.04.2021	20	0
Finland	11.04.2021	21	23
France	11.04.2021	101	3
Germany	11.04.2021	78	6
Greece	11.04.2021	87	0
Italy	12.04.2021	178	31
Malta	12.04.2021	9	0
Netherlands	12.04.2021	3	0
Poland	12.04.2021	144	4
Portugal	12.04.2021	35	0
Russia	12.04.2021	28	14
Serbia	12.04.2021	179	0
Spain	12.04.2021	570	227
Sweden	12.04.2021	1	0
United Kingdom	12.04.2021	6	8

## 2 Experimental Design, Materials and Methods

Cobalt's multiple alignment output for each European country was processed by C# application developed in Visual Studio 2019 and thus dataset presented in this article was generated. The list of mutations was recorded in string format. C# program performed residues’ comparison between YP_009724390.1 reference and each SARS-CoV-2 spike protein to identify mutations. For that reason, string variable Changes was used. Each time new SARS-CoV-2 spike protein was about to be processed, (string) Changes was set up to empty string “”. As mutations were detected, they were tracked to (string) Changes in: (X→Y)p:P or (X→−)p:P form, depending of the type of mutation. Once the protein was processed, record: $\left(\text{string}\right)\;\text{Changes}=\left(X_{1}\to Y_{1}\right)\;p:\;P_{1}\;\left(X_{2}\to Y_{2}\right)\;p:\;P_{2}\;\left(X_{3}\to -\right)\;p:\;P_{3}\ldots$ was obtained. Delimiter empty space was used to sperate individual mutations. The following code summarizes this discussion.

FOREACH European country

RUN Cobalt for YP_009724390.1 AND SARS-CoV-2 spike proteins

FROM Cobalt output:

FOREACH SARS-COV-2 spike protein:

(string) Changes=””

FOR(i=0;i<SARS-COV-2 spike protein.Length;i++)

IF(YP_009724390.1[i]!=SARS-COV-2 spike protein[i])

Changes+=YP_009724390.1[i]+”->”+SARS-COV-2 spike protein[i]+” p: ”+i.ToString()+” ”

File.Write(”Seq: ID:”+spike protein NCBI accession id+”, Changes: ”+Changes)

**Fig. 2:**
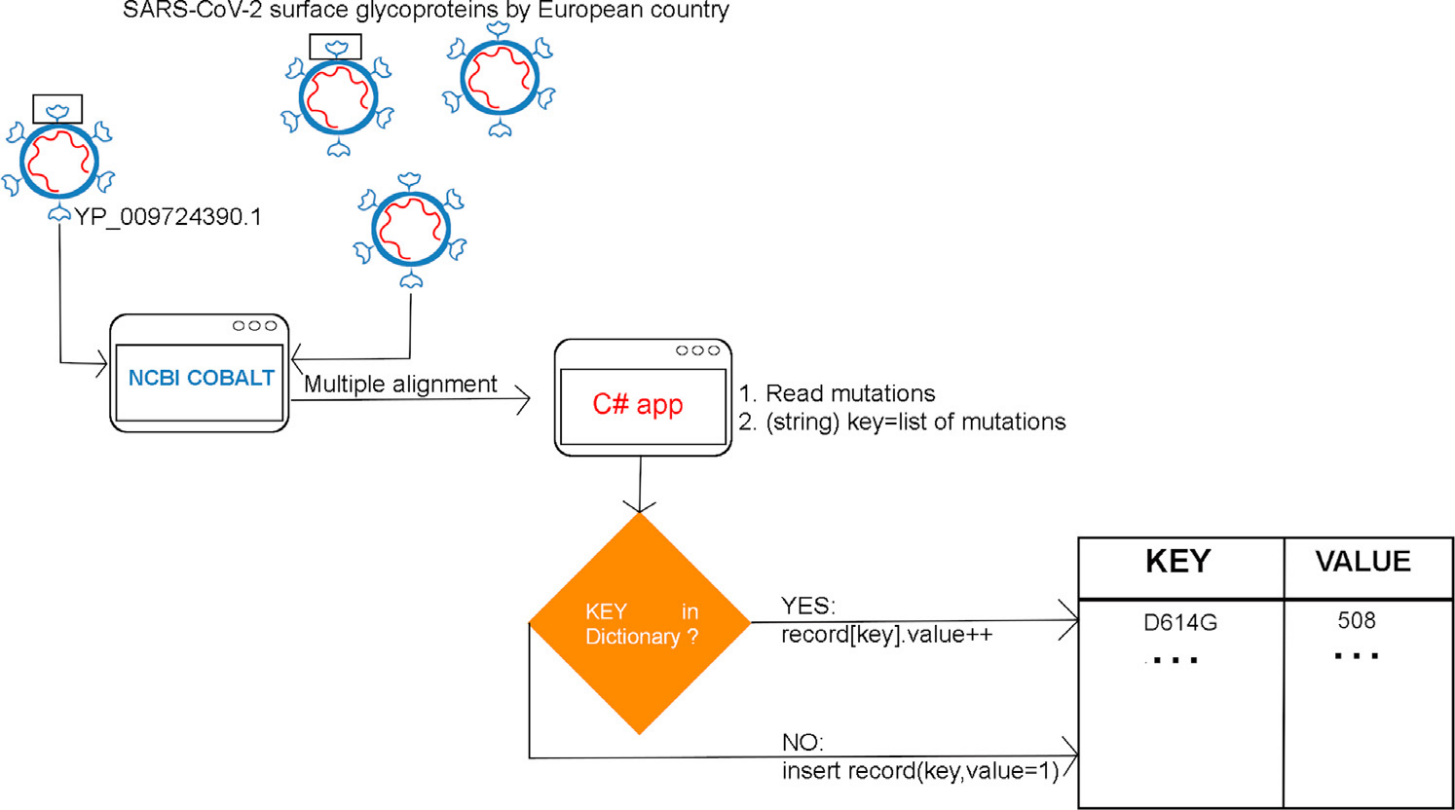
Computing SARS-CoV-2 spike protein variants in Europe.

Mutations reports available in dataset reported in this article, served as a source to compute SARS-CoV-2 spike protein variants reported in Appendix A in [2]. For that reason, Dictionary<string, int> was used, Fig. 2. Each dictionary entry is regarded as a (string, int) tuple of key and value, such as: (string) key tracks mutations as: XpY or Xp− (depending of the type) and (int) value tracks the number of SARS-CoV-2 spike proteins matching specific key, Fig. 2. Before processing the spike protein, text variable key is initialized to an empty string (“”). As mutations are identified, based on residues’ comparison, they are appended to the key, such as $\left(\text{string}\right)\;\text{key}=X_{1}p_{1}Y_{1}X_{2}p_{2}Y_{2}X_{3}p_{3}-\ldots$ is obtained, Fig. 2.

If the computed key is not contained in the dictionary, then entry: (key, value=1) is added to the dictionary, Fig. 2. Otherwise, given that the computed key is already contained in the dictionary, then (int) value is increased for one, Fig. 2. This scenario occurs when: second, third…etc SARS-CoV-2 spike protein with the same combination of mutations is identified and given that the key tracking that specific combination of mutations is already stored in the dictionary, the number of SARS-CoV-2 spike proteins matching that specific variant: (int) value needs to be increased for one, each time the same combination of mutations is identified.

For this reason, the previous code was upgraded up to variants’ tracking ability at continental level. The upgrade is suggested by the code in italic bellow. The whole process is clearly illustrated on Fig. 2.

*Dictionary<string, int> Variants=empty Dictionary*

FOREACH European country

RUN Cobalt for YP_009724390.1 AND SARS-CoV-2 spike proteins

FROM Cobalt output:

FOREACH SARS-COV-2 spike protein:

(string) Changes=””

*(string) key=””*

FOR(i=0;i<SARS-COV-2 spike protein.Length;i++)

IF(YP_009724390.1[i]!=SARS-COV-2 spike protein[i])

Changes+=YP_009724390.1[i]+”->”+SARS-COV-2 spike protein[i]+” p: ”+i.ToString()+” ”

*key+=YP_009724390.1[i]+i.ToString()+SARS-COV-2 spike protein[i]*

File.Write(”Seq: ID:”+spike protein NCBI accession id+”, Changes: ”+Changes)

*IF(Variants.ContainsKey(key))*

*Variants[key].value++*

*Else*

*Variants.Add(key,value=1)*

## CRediT Author Statement

**Done Stojanov:** Conceptualization, Methodology, Data Collection, Visualization, Writing-Original draft preparation.

## Declaration of Competing Interest

The author declare no known competing financial interests or personal relationships that could have appeared to influence the work reported in this paper.

## Appendix Supplementary materials

Image, application 1
